# PM2.5 Induced the Expression of Fibrogenic Mediators via HMGB1-RAGE Signaling in Human Airway Epithelial Cells

**DOI:** 10.1155/2018/1817398

**Published:** 2018-01-28

**Authors:** Weifeng Zou, Fang He, Sha Liu, Jinding Pu, Jinxing Hu, Qing Sheng, Tao Zhu, Tianhua Zhu, Bing Li, Pixin Ran

**Affiliations:** ^1^The State Key Laboratory of Respiratory Disease, Guangzhou Chest Hospital, Guangzhou, Guangdong, China; ^2^The Research Center of Experiment Medicine, Guangzhou Medical University, Guangzhou, Guangdong, China; ^3^The State Key Laboratory of Respiratory Disease, Guangzhou Institute of Respiratory Diseases, The First Affiliated Hospital, Guangzhou Medical University, Guangzhou, Guangdong, China; ^4^The Third Affiliated Hospital of Guangzhou Medical University, Guangzhou, Guangdong, China

## Abstract

**Background:**

The aim of the present study was to test whether fine particulate matter (PM2.5) induces the expression of platelet-derived growth factor-AB (PDGF-AB), PDGF-BB, and transforming growth factor-*β*1 (TGF-*β*1) in human bronchial epithelial cells (HBECs) in vitro via high-mobility group box 1 (HMGB1) receptor for advanced glycation end products (RAGE) signaling.

**Methods:**

Sprague-Dawley rats were exposed to motor vehicle exhaust (MVE) or clean air. HBECs were either transfected with a small interfering RNA (siRNA) targeting HMGB1 or incubated with anti-RAGE antibodies and subsequently stimulated with PM2.5.

**Results:**

The expression of HMGB1 and RAGE was elevated in MVE-treated rats compared with untreated rats, and PM2.5 increased the secretion of HMGB1 and upregulated RAGE expression and the translocation of nuclear factor κB (NF-κB) into the nucleus of HBECs. This activation was accompanied by an increase in the expression of PDGF-AB, PDGF-BB, and TGF-*β*1. The HMGB1 siRNA prevented these effects. Anti-RAGE antibodies attenuated the activation of NF-*κ*B and decreased the secretion of TGF-*β*1, PDGF-AB, and PDGF-BB from HBECs.

**Conclusion:**

PM2.5 induces the expression of TGF-*β*1, PDGF-AB, and PDGF-BB in vitro via HMGB1-RAGE signaling, suggesting that this pathway may contribute to the airway remodeling observed in patients with COPD.

## 1. Introduction

Chronic obstructive pulmonary disease (COPD) is characterized by partially reversible air-flow obstruction that is frequently ascribed to airway remodeling. PM2.5 (particles with an aerodynamic diameter less than 2.5 *μ*m) have been associated with an increased risk of COPD, respiratory symptoms, and impaired lung function in epidemiological studies [1–3].

High-mobility group box 1 (HMGB1) is a ubiquitous nuclear protein that acts as a crucial factor in acute lung injury progression and pulmonary fibrosis [4]. HMGB1 is actively or passively released from various cells in response to stimulation with endogenous proinflammatory cytokines [5]. The receptors for HMGB1 include receptor for advanced glycation products (RAGE), Toll-like receptor 2 (TLR2), and TLR4 [6]. Elevated HMGB1 expression in COPD airways might sustain inflammation and remodeling through interactions with RAGE [7]. The interaction of RAGE with its ligand HMGB1 induces the production of a number of profibrotic cytokines, such as PDGF and TGF-*β*1, in the lungs [8]. TGF-*β*1 and PDGF are among the most important fibrogenic mediators that participate in the pathophysiology of pulmonary fibrosis.

To our knowledge, no study has systematically examined the effects of PM2.5 on human bronchial epithelial cells (HBECs) in vitro and the possible molecular mechanisms that regulate the expression of TGF-*β*1 and PDGF. According to epidemiological studies, PM2.5 might regulate the expression of HMGB1-RAGE signaling intermediates and associated mechanisms in elderly men [9]. Thus, in the present study, we investigated whether PM2.5 induces the expression of TGF-*β*1 and PDGF in HBECs in vitro via HMGB1-RAGE signaling.

## 2. Materials and Methods

### 2.1. Materials

DMEM and fetal bovine serum (FBS) were purchased from Sigma Chemical Co. (St. Louis, MO, USA). The anti-NF-kB p65 antibody, anti-HMGB1 antibody, HMGB1 siRNA (h), and the siRNA transfection reagent were purchased from Santa Cruz Biotechnology (Santa Cruz, CA, USA). The anti-TLR4 antibody, neutralizing anti-RAGE antibody, isotype-matched control antibody (IgG), and TGF-*β*1, PDGF-AB, and PDGF-BB ELISA kits were obtained from R&D Systems (Minneapolis, MN, USA). The anti-TLR2 antibody was purchased from Novus Biologicals (Novus, CO, USA). The HMGB1 ELISA kit was purchased from Shino-Test (Tokyo, Japan).

### 2.2. Animals

Eighteen female Sprague-Dawley rats (body weight 180–200 g, 6–8 weeks old) were socially housed (up to four rats per cage) in the Laboratory Animal Center of the First Affiliated Hospital, Guangzhou Medical University, which approved the use of experimental animals in this study. The rats were randomly divided into a MVE group and a clean air control group. The rats in the MVE group were exposed to MVE (1.5 mg/m^3^) for 2 h periods, 5 days per week for 1 month. In the MVE exposure room, the concentrations of PM2.5, PM10, and PM1 were 1.46 ± 0.034 mg/m^3^, 1.47 ± 0.034 mg/m^3^, and 1.45 ± 0.035 mg/m^3^, respectively. The O_2_, CO, NO_1_, NO_X_, and SO_2_ levels in the exposure rooms were 20.95 ± 0.006%, 67.524 ± 3.565 ppm, 0.50 ± 0.211 ppm, 0.50 ± 0.211 ppm, and 0.35 ± 0.181 ppm, respectively [10].

### 2.3. Sampling Lung Tissues

Rats were anesthetized via an intraperitoneal injection of 3% pentobarbital (1 ml/kg). The anesthesia was maintained at a light surgical plane for the duration of testing. Rats were sacrificed by intraperitoneal injection of sodium pentobarbital (100 mg/kg). The left or right lung was inflated and fixed using 4% paraformaldehyde (pH 7.40) at 25 cmH_2_O pressure for 24 h. The lungs were then embedded in paraffin and cut into 4 *μ*m thick sections [10].

### 2.4. Collection and Extraction of PM2.5

Briefly, traffic ambient PM2.5 was collected by aerodynamic impactors equipped with a glass fiber filter, quartz filter, or teflon membrane, depending on the specific purpose. Four batches of PM2.5 filter samples were extracted with DMSO. Mean concentrations of traffic ambient PM2.5 in spring and summer were 79.95 ± 23.89 *μ*g/m^3^ and 46.29 ± 12.87 *μ*g/m^3^, respectively. Mean concentrations of these extracts were 11.96 ± 0.96 mg/ml, whereas the recovery from a single membrane was 90.7% ± 5.9%. Subsequently, the quantification and characterization of PM2.5, including polynuclear aromatic hydrocarbons (PAHs), *n*-alkanes, metals, and water-soluble inorganic ions, were performed using a gravimetric analysis, thermal desorption-gas chromatography-mass spectrometry (TD-GC-MS), energy-dispersive X-ray fluorescence (ED-XRF) spectrometry, and ion chromatography (IC). The mean concentration of PAHs in PM2.5 was 108.453 *μ*g/g, and the concentration in DMSO extracts was 48.392 *μ*g/g, with a final PAH recovery of 44.62%. The mean concentration of *n*-alkanes in PM2.5 was 18,670.883 *μ*g/g. The DMSO extracts had a concentration of 164.675 *μ*g/g [11, 12].

### 2.5. Cell Culture

Human bronchial epithelial cells (HBECs; ATCC) were cultured in 2 ml of DMEM containing 10% FBS and penicillin (100 U/ml) at a density of 5-6 × 10^5^ cells per 6-well plate. Prior to the experiments, the cells were serum starved for 24 h, transfected with the HMGB1 siRNA or control siRNA for 48 h or pretreated with anti-RAGE antibodies (10 g/ml) and IgG for 1 h, and subsequently stimulated with 5–20 *μ*g/ml of PM2.5 in medium for 12–48 h. The cells were then harvested for further analyses.

### 2.6. Small Interfering RNA Preparation and Transfection

One day prior to transfection, the cells were plated in growth medium without antibiotics and cultured until they reached 60–80% confluency at the time of transfection. The cells were transfected with 80 pmol/L siRNA duplexes (control or HMGB1) using a transfection reagent and transfection medium, according to the manufacturer's instructions.

### 2.7. Real-Time Quantitative Polymerase Chain Reaction (PCR)

Total RNA was prepared from cells using the RNeasy plus mini kit (Qiagen), according to the manufacturer's instructions, with the following sequence-specific primers: RAGE, 5′-GAAAGCCCTCCTGTCAGCATC-3′ and 5′-GGCACCATTCTCTGGCA TCTC-3′; TLR-2, 5′-CTTCCAGGTCTTCAGTCTTC-3′ and 5′-TGA TTGCGGACACATCTC-3′; TLR-4, 5′-GCCGTTGGTGTATCTTTG-3′ and 5′-GCTGTTTGCTCAGGATTC-3′; and GAPDH, 5′-ATCACTGCCACCC AGAAG-3′ and 5′-TCCACGACGGACACATTG-3′. Quantity RT-PCR reactions were performed with an MXP3000 QPCR system (Stratagene, USA) under the following conditions: 30 s at 95°C, 40 cycles of 5 s at 95°C and 30 s at 60°C, 1 min at 95°C, and an increase from 60°C to 95°C. The housekeeping gene *GAPDH* was used as an internal control. The data were normalized to GAPDH levels and expressed as a fold change relative to the control.

### 2.8. Extraction of Cytoplasmic and Nuclear Proteins and Western Blotting

The cells were lysed and incubated in a cytoplasmic extract buffer. The remaining nuclei were washed and resuspended in nuclear extract buffer. Protein concentrations were quantified using the bicinchoninic acid method. Subsequently, the proteins were separated by SDS-PAGE, transferred to polyvinylidene difluoride membranes, and probed with a rabbit polyclonal anti-RAGE antibody (1 : 1000), mouse polyclonal anti-TLR2 antibody (1 : 1000), mouse polyclonal anti-TLR4 antibody (1 : 1000), or mouse polyclonal anti-NF-*κ*B p65 antibody (1 : 1000). Antibody binding was detected using chemiluminescence according to the manufacturer's instructions. GAPDH (1 : 5000), *β*-tubulin (1 : 2000), and lamin B (1 : 500) antibodies were used as loading controls. The bands obtained for HMGB1 receptors and cytosolic and nuclear levels of NF-*κ*B were normalized to GAPDH, *β*-tubulin, and lamin B, respectively, and expressed as a fold change relative to the control.

### 2.9. Immunofluorescence

Cells were seeded on sterile round coverslips placed in 12-well plates. PM2.5 was added to a subset of wells at a final concentration of 20 *μ*g/ml. Forty-eight hours later, the cells were stained with the anti-RAGE antibody (1 : 50) for 1 h at room temperature. Antibody binding was detected using a peroxidase-conjugated anti-mouse or anti-rabbit antibody, according to the manufacturer's instructions. Cell nuclei were stained with 4,6-diamidino-2-phenylindole (DAPI). Immunofluorescence was examined using a Leica confocal microscope at ×400 magnification.

### 2.10. Immunohistochemistry

Paraffin sections of lung samples were stained with antibodies against HMGB1 and RAGE for 1 h at room temperature. HMGB1 and RAGE (1 : 50) antibody binding was detected using a peroxidase-conjugated anti-mouse or anti-rabbit antibody and DAB, according to the manufacturer's instructions. Expression was visualized using a confocal microscope at ×400 magnification. We determined the optical density (OD) of HMGB1- and RAGE-positive cells using a semiquantitative scoring system.

### 2.11. ELISA

Cell supernatants were collected, and HMGB1, TGF-*β*1, and PDGF levels were measured using HMGB1, TGF-*β*1, and PDGF ELISA kits, respectively, according to the manufacturer's instructions. Samples with concentrations exceeding the standard curve limits were diluted until an accurate reading was obtained. Four replicate wells were used to obtain all data points, and all samples were processed in duplicate and averaged.

### 2.12. Statistical Analysis

Data from at least 3 independent sets of experiments were analyzed using the SPSS 17.0 statistical software and expressed as means + SD. Statistical evaluations of continuous data were performed using ANOVA or the independent samples *t*-test for between-group comparisons. The level of significance was set to *P* < 0.05.

## 3. Results

### 3.1. HMGB1 and RAGE Expression in the Representative Lung Tissue

**Figure 1:**
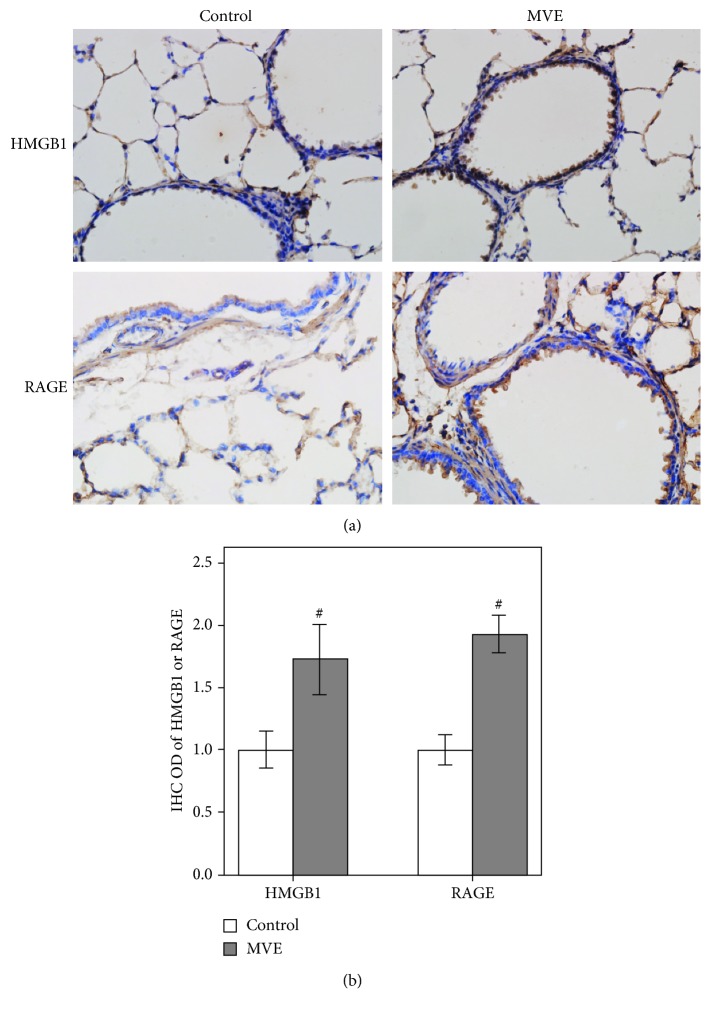
HMGB1 and RAGE expression in bronchial biopsies and in lung tissue sections. (a) Immunohistochemical staining showing an increase in HMGB1 and RAGE staining in MVE-treated rats compared with untreated rats. The HMGB1 protein was primarily located in the nucleus and cytoplasm of epithelial cells. RAGE was expressed in the cytoplasm of bronchial epithelial cells and alveolar epithelial cells; magnification ×400. (b) The ODs of HMGB1- and RAGE-positive cells were higher in MVE-treated rats than in untreated rats.  ^#^*P* < 0.05, compared with the control group.

The distribution of HMGB1 and RAGE in lung tissue sections from experimental Sprague-Dawley rats was determined by immunostaining. A large number of HMGB1- and RAGE-positive bronchial epithelial cells and alveolar epithelial cells were detected in MVE-treated rats compared with untreated rats (Figures 1(a) and 1(b)). Because the translocation of HMGB1 from the nucleus to the cytoplasm is considered a hallmark of the active secretion of this protein in the extracellular milieu [13], we also determined the subcellular localization of HMGB1 and RAGE. The HMGB1 protein was primarily located in the nucleus and cytoplasm of epithelial cells. RAGE was expressed in the cytoplasm of bronchial epithelial cells and alveolar epithelial cells (Figure 1(a)). Thus, MVE induced the expression of HMGB1 and RAGE in rats, and these cells represent a potential source of the secreted soluble form of HMGB1 in the airways.

### 3.2. PM2.5 Induces the Secretion of HMGB1 and Upregulates RAGE Expression in HBECs

**Figure 2:**
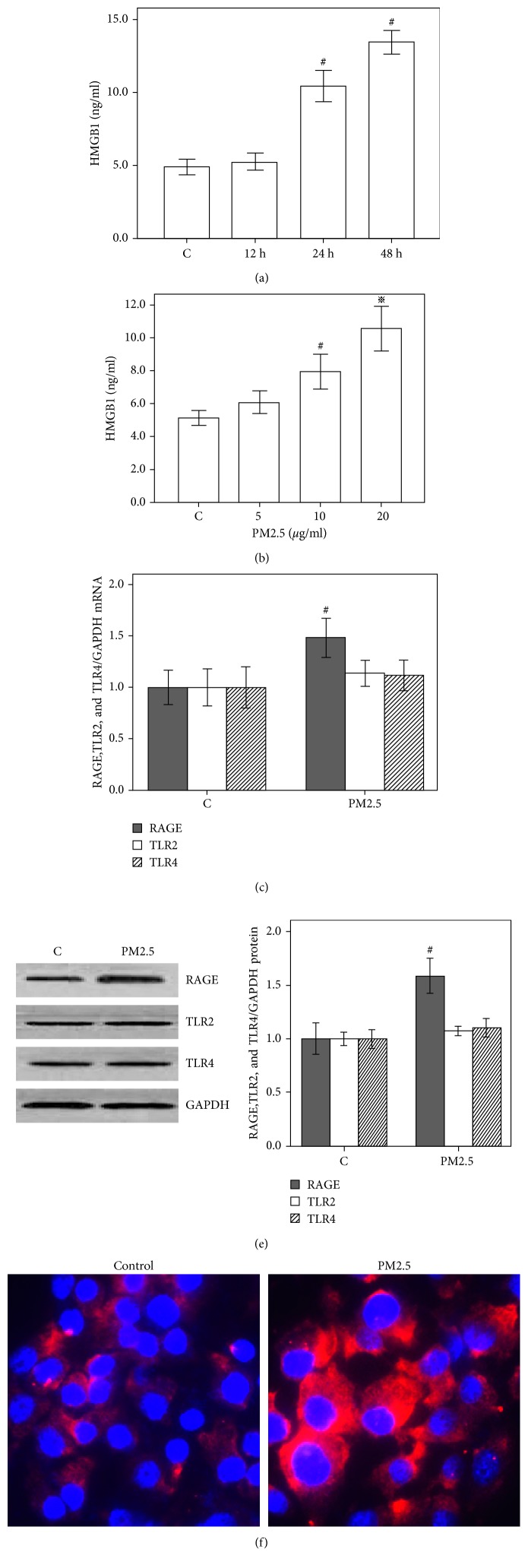
PM2.5 induces HMGB1 secretion and upregulates RAGE expression in HBECs. HBECs were incubated with PM2.5 for 12, 24, and 48 h. (a) Based on the ELISA results, HMGB1 secretion was increased upon stimulation with PM2.5 (20 *μ*g/ml) for 24 and 48 h; no significant changes were observed at 12 h. (b) ELISA results show that HMGB1 expression increases 48 h after PM2.5 exposure in a concentration-dependent manner. (c) Real-time quantitative PCR analysis showing an increase in the expression of the RAGE mRNA after 48 h of exposure to PM2.5 (20 *μ*g/ml); no significant differences were observed in the levels of the TLR2 and TLR4 mRNAs. These data show 1.48-fold (RAGE), 1.14-fold (TLR2), and 1.12-fold (TLR4) increases compared with the untreated control, respectively, using the GAPDH mRNA for calibration. (d) Western blot analysis showing that PM2.5 (20 *μ*g/ml) increases the levels of the RAGE protein, with no significant changes in the levels of the TLR2 and TLR4 proteins. (e) Immunofluorescence staining shows that PM2.5 (20 *μ*g/ml) affects the levels of the RAGE protein. The RAGE/GAPDH ratio in control cells is set to 1.  ^#^*P* < 0.05, compared with the control group.  ^※^*P* < 0.05, compared with the PM2.5 group, *n* = 3.

Based on our observations, PM2.5 was one of the main components detected in the PM exposure room; therefore, we endeavored to further assess whether PM2.5 induced HMGB1 secretion and upregulated the expression of its receptors in HBECs. According to the ELISA data, the cells did not show significant changes in HMGB1 secretion after 12 h of PM2.5 (20 g/ml) stimulation, but the levels were significantly increased at 24 and 48 h compared with the baseline levels (Figure 2(a)). A 48 h exposure to PM2.5 (5–20 g/ml) increased HMGB1 secretion from HBECs in a concentration-dependent manner (Figure 2(b)). Moreover, the relative levels of the RAGE mRNA were increased after 48 h of PM2.5 treatment, but significant differences in the levels of TLR2 and TLR4 were not detected, although a slight increase in expression was observed (Figure 2(c)). Western blot data were consistent with the qRT-PCR data (Figure 2(d)), and as shown in Figure 2(e), PM2.5 induced RAGE expression.

### 3.3. The Role of HMGB1 in the PM2.5-Induced Expression of HMGB1 Receptors in HBECs

**Figure 3:**
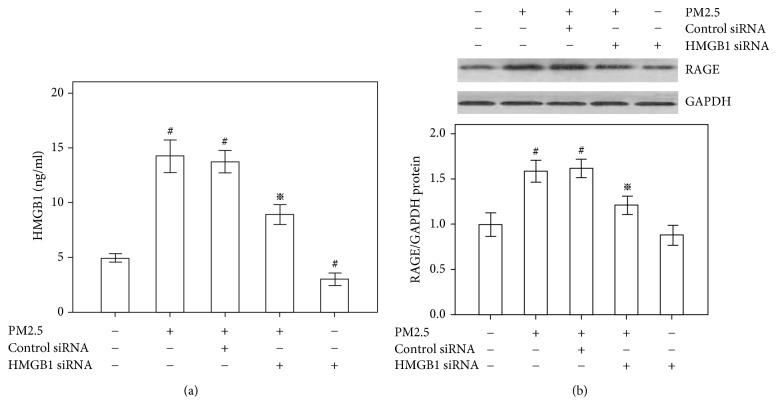
Role of HMGB1 in the PM2.5-induced expression of RAGE in HBECs. Cells were transfected with the HMGB1 siRNA or control siRNA for 48 h and subsequently stimulated with PM2.5 (20 *μ*g/ml) for 48 h. (a) According to the ELISA results, HMGB1 knockdown reduces HMGB1 secretion in the presence of PM2.5. (b) Western blot showing that HMGB1 knockdown reduces RAGE expression in the presence of PM2.5. The RAGE/GAPDH ratio in control cells is set to 1.  ^#^*P* < 0.05, compared with the control group.  ^※^*P* < 0.05, compared with the PM2.5 group, *n* = 3.

We used siRNAs to deplete HMGB1 expression and further assess the extracellular effect of HMGB1. Cells were transfected with an HMGB1 siRNA or control siRNA for 48 h and subsequently stimulated with PM2.5 (20 *μ*g/ml) for 48 h. The transfection of the HMGB1 siRNA into the cells significantly decreased HMGB1 production in both PM2.5-treated and untreated cells. Compared to the negative control siRNA, which did not inhibit the enhanced HMGB1 production observed after PM2.5 treatment, the HMGB1 siRNA transfection suppressed the increase in HMGB1 production; however, HMGB1 production was higher than the untreated control (Figure 3(a)). Cells transfected with the HMGB1 siRNA showed decreased RAGE production compared with cells stimulated with PM2.5 (Figure 3(b)), indicating that the upregulation of RAGE in HBECs exposed to PM2.5 was mediated by HMGB1.

### 3.4. HMGB1-RAGE Signaling Contributes to PM2.5-Induced NF-*κ*B Activity in HBECs

**Figure 4:**
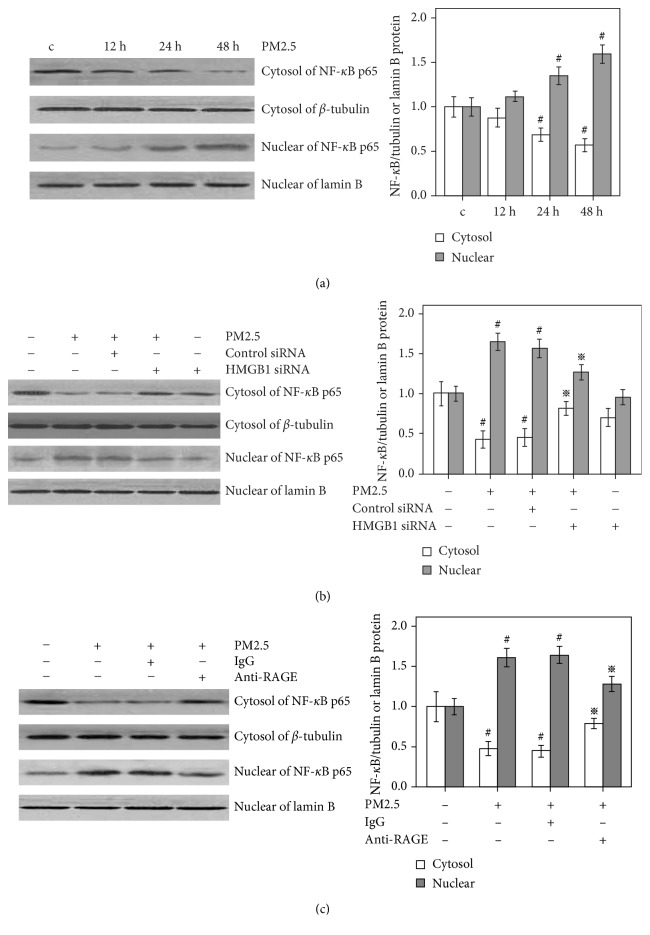
Effects of PM2.5 and HMGB1-RAGE signaling on NF-*κ*B activity in HBECs. Cells were stimulated with PM2.5 (20 *μ*g/ml) for 12, 24, and 48 h. (a) Western blot analysis showing that PM2.5 decreased the cytosolic levels of NF-*κ*B and increased the nuclear levels after 24 and 48 h of PM2.5 stimulation; no significant difference was observed at 12 h of PM2.5 stimulation. (b) Cells were transfected with the HMGB1 siRNA or control siRNA for 48 h and subsequently stimulated with PM2.5 (20 *μ*g/ml) for 48 h. Western blot analysis showing that cells transfected with the HMGB1 siRNA increased the cytosolic levels of NF-*κ*B and attenuated nuclear NF-*κ*B levels compared with cells stimulated with PM2.5. (c) Cells were pretreated with anti-RAGE antibodies (10 *μ*g/ml) or IgG for 1 h and subsequently exposed to PM2.5 (20 *μ*g/ml) for 48 h. Western blot analysis showing that cell pretreated with anti-RAGE antibodies increased the cytosolic levels of NF-*κ*B and attenuated nuclear NF-*κ*B levels compared with cells stimulated with PM2.5. The cytosolic NF-*κ*B/tubulin and nuclear NF-*κ*B/lamin B ratio of the control cells is set to 1.  ^#^*P* < 0.05, compared with the control group.  ^※^*P* < 0.05, compared with the PM2.5 group, *n* = 3.

We characterized the potential downstream signaling events in cells with increased RAGE expression. Accordingly, western blot results did not reveal significant changes in NF-*κ*B levels after 12 h of PM2.5 stimulation compared with baseline levels, but significantly decreased cytosolic levels of NF-*κ*B and increased nuclear levels were observed at 24 and 48 h of PM2.5 stimulation (Figure 4(a)). Cells transfected with the HMGB1 siRNA showed increased cytosolic levels of NF-*κ*B and attenuated nuclear NF-*κ*B levels compared with cells stimulated with PM2.5 (Figure 4(b)). Furthermore, cells were pretreated with anti-RAGE antibodies and IgG for 1 h and subsequently exposed to PM2.5 (20 *μ*g/ml) for 48 h. Anti-RAGE antibodies significantly increased the cytosolic levels of NF-*κ*B and decreased the nuclear levels of NF-*κ*B (Figure 4(c)) compared to the levels observed when cells were pretreated with a negative control antibody (IgG), indicating that HMGB1-RAGE signaling was involved in PM2.5-induced NF-*κ*B activation.

### 3.5. The Role of HMGB1-RAGE Signaling in PM2.5-Induced TGF-*β*1 and PDGF Production in HBECs

**Figure 5:**
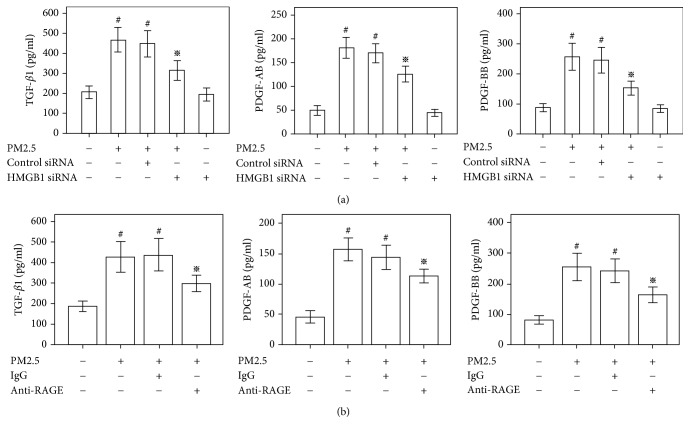
Role of HMGB1 in PM2.5-induced TGF-*β*1 and PDGF production in HBECs. Cells were transfected with the HMGB1 siRNA or control siRNA for 48 h and subsequently stimulated with PM2.5 (20 *μ*g/ml) for 48 h. (a) According to the ELISA results, PM2.5 induces the secretion of TGF-*β*1, PDGF-AB, and PDGF-BB. HMGB1 knockdown reduces the secretion of TGF-*β*1, PDGF-AB, and PDGF-BB in the presence of PM2.5. (b) Cells were pretreated with anti-RAGE antibodies or IgG for 1 h and subsequently exposed to PM2.5 (20 *μ*g/ml) for 48 h. According to the ELISA results, anti-RAGE antibodies reduce the secretion of TGF-*β*1, PDGF-AB, and PDGF-BB in the presence of PM2.5.  ^#^*P* < 0.05, compared with the control group.  ^※^*P* < 0.05, compared with the PM2.5 group, *n* = 3.

We examined the secretion of TGF-*β*1 and PDGF in HBECs cultured with PM2.5 using ELISAs to determine the molecular mechanism by which PM2.5 activated HBECs. Based on the ELISA data, the level of the TGF-*β*1 protein was increased in these cells after a 48 h exposure to PM2.5 (Figures 5(a) and 5(b)). Dimeric isoforms of PDGF-A and PDGF-B chains, such as PDGF-AB and PDGF-BB, play important roles in the pathogenesis of fibrosis. A 48 h exposure to PM2.5 also increased PDGF-AB and PDGF-BB production in HBECs (Figures 5(a) and 5(b)). Cells transfected with the HMGB1 siRNA or incubated with the anti-RAGE antibody showed decreased secretion of the profibrotic cytokines TGF-*β*1, PDGF-AB, and PDGF-BB compared with cells stimulated with PM2.5 (Figures 5(a) and 5(b)). Based on these results, PM2.5 induces TGF-*β*1 and PDGF production in HBECs through an HMGB1-RAGE-dependent mechanism.

## 4. Discussion

Many studies have reported associations between air pollution and the exacerbation of preexisting COPD [14], although tobacco smoking is the primary cause of COPD. Many other environmental and occupational exposures contribute to its pathology. As shown in our previous study, MVE exposure causes airway cells to release multiple cytokines that are capable of inducing pronounced COPD in rats [10]. In the present study, a large number of HMGB1- and RAGE-positive bronchial epithelial cells and alveolar epithelial cells were detected in MVE-treated rats compared with untreated rats. These cells represent a potential source of the secreted soluble form of HMGB1 in the airways. These results are consistent with a previous study showing that RAGE and HMGB1 mRNA levels were increased in rats exposed to ozone plus diesel exhaust particulate [15]. PM2.5 was one of the main components observed in the PM exposure room. Combined with the results from our previous study, the results from the present study provided the first evidence that 48 h of PM2.5 stimulation induces HMGB1 secretion, upregulates RAGE expression, and promotes the translocation of NF-*κ*B to the nucleus of HBECs.

Moreover, PM2.5 induced the production of the profibrotic cytokines TGF-*β*1, PDGF-AB, and PDGF-BB in HBECs. HMGB1-RAGE signaling increases the levels of profibrotic cytokines, such as TGF-*β*1, and PDGF, in the lungs [8]. HBECs express TLR2, TLR4, and RAGE, which bind HMGB1 [16], indicating that HBECs respond to HMGB1. Furthermore, HMGB1-RAGE signaling might also be involved in mechanisms related to PM2.5 in vivo [9]. Based on experimental data, HMGB1 activates RAGE signaling and induces NF-*κ*B activation to promote cellular proliferation in hepatocellular carcinoma (HCC) cell lines [17]. Thus, HBECs stimulated with PM2.5 exhibited increased expression of extracellular HMGB1 and RAGE, accompanied by the nuclear translocation of NF-*κ*B and production of TGF-*β*1, PDGF-AB, and PDGF-BB, which are critical factors involved in the development of fibrosis and airway remodeling.

HMGB1 alone increases the expression of proinflammatory cytokines in HBECs, primarily through RAGE [18]. We transfected cells with an HMGB1 siRNA to block the PM2.5-induced expression of cytokines and HMGB1 receptors. Transfection with the HMGB1 siRNA alone significantly inhibited the PM2.5-induced secretion of TGF-*β*1, PDGF-AB, and PDGF-BB, decreased RAGE production, and attenuated the activation of NF-*κ*B, strongly suggesting that HMGB1 is primarily involved in the PM2.5-induced production of the profibrotic cytokines TGF-*β*1, PDGF-AB, and PDGF-BB. Mechanistically, NF-*κ*B signaling has been shown to be important in HMGB1-induced synovial fibroblast migration, is associated with abnormal proliferation of cells in the lungs [19], and is significantly involved in mediating PDGF-BB and TGF-*β*1 production [20, 21].

In addition, RAGE is the main receptor mediating the effects of HMGB1 on profibrotic cytokines in HBECs. HMGB1 itself enhances the production of proinflammatory cytokines through RAGE in the absence of TLR activation [18]. Consistent with the results from these reports, upregulation of RAGE and the nuclear translocation of NF-*κ*B in cells exposed to PM2.5 were mediated by HMGB1 in our study. Cellular signaling through RAGE has been suggested to play a role in DPM- (diesel particulate matter-) induced NF-*κ*B activation and chemokine responses in a type-I-like epithelial cell line [22]. However, HMGB1 has been reported to increase cytokine production in macrophages in response to proinflammatory cytokines through TLR2 and TLR4 [23]. Based on this evidence, we used anti-RAGE antibodies to block the expression of PM2.5-induced cytokines and activate NF-*κ*B. RAGE influenced NF-*κ*B activation and the production of the profibrotic cytokines TGF-*β*1, PDGF-AB, and PDGF-BB following PM2.5 stimulation, suggesting that PM2.5 induces the expression of extracellular HMGB1 that in turn upregulates RAGE expression, which participates in the activation of NF-*κ*B to induce the production of TGF-*β*1, PDGF-AB, and PDGF-BB.

In summary, we reported previously unknown findings showing that HMGB1 and RAGE expression was elevated in MVE-treated rats compared with untreated rats. PM2.5 induced the secretion of HMGB1 and promoted TGF-*β*1, PDGF-AB, and PDGF-BB production in HBECs in a RAGE-dependent manner, and this mechanism is partially dependent on NF-*κ*B activation. These results will contribute to a better understanding of the airway remodeling occurring in patients with COPD who are exposed to PM2.5 and the development of new therapeutic approaches.
